# Fascial Nomenclature: An Update

**DOI:** 10.7759/cureus.5718

**Published:** 2019-09-21

**Authors:** Bruno Bordoni, Stevan Walkowski,, Bruno Morabito, Matthew A Varacallo

**Affiliations:** 1 Cardiology, Foundation Don Carlo Gnocchi, Milan, ITA; 2 Osteopathic Manipulative Medicine, Heritage College of Osteopathic Medicine-Dublin, Ohio, USA; 3 Osteopathy, School of Osteopathic Centre for Research and Studies, Milan, ITA; 4 Orthopaedic Surgery and Sports Medicine, University of Kentucky, Lexington, USA

**Keywords:** fascia, myofascial, osteopathic, physiotherapy, mechanotransduction, skeletal muscle

## Abstract

Throughout the development of anatomy as a scientific study, authors have been challenged to give a singular comprehensive definition of what should be considered as a fascial tissue. Instead, the multiplicity of synthesis and analysis is the true richness of scientific research: individual points of view and background look at the fascia from their own perspective, sometimes influenced by their own cultural assumptions. No person or organization in science ever have the absolute truth, because scientific truth is always evolving, driven by new observations and analysis of data. Only by observing the fascia from multiple perspectives (doctor, surgeon, osteopath, physiotherapist, bioengineer and more) can we define more fully what fascial tissue is. It becomes the synergistic result of several scientific disciplines (anatomy, cardiology, angiology, orthopaedics, osteopathy, cytology, and more). The fascia is not the exclusive domain of a few people or individual private associations, but of all researchers who journey through the study of knowledge and arrive at an understanding, improving the clinical aspects for the good of the patient, without profit. This article reviews the embryological evolution of muscle and connective tissue to affirm how the fascial system should be ideally conceptualized: an absolute anatomic functional continuum.

## Introduction and background

Research should always be free from lucrative financial intentions, just as researchers should not have the objective of earning money by limiting the knowledge and clinical application of information to the exclusion of other health professionals. According to the Foundation of Osteopathic Research and Clinical Endorsement (FORCE), an international research group founded in 2013 and comprised of different professional disciplines (surgery, osteopathy, physiotherapy, bio-engineering, etc.), has recently disclosed what should be considered as fascial tissue, taking into account the most current scientific information: “The fascia is any tissue that contains features capable of responding to mechanical stimuli. The fascial continuum is the result of the evolution of the perfect synergy among different tissues, liquids and solids, capable of supporting, dividing, penetrating, feeding and connecting all the regions of the body, from the epidermis to the bone, involving all its functions and organic structures. This continuum constantly transmits and receives mechanometabolic information that can influence the shape and function of the entire body. These afferent/efferent impulses come from the fascia and the tissues that are not considered as part of the fascia in a biunivocal mode. In this definition, these tissues include: epidermis, dermis, fat, blood, lymph, blood and lymphatic vessels, tissue covering the nervous filaments (endoneurium, perineurium, epineurium), voluntary striated muscle fibers and the tissue covering and permeating it (epimysium, perimysium, endomysium), ligaments, tendons, aponeurosis, cartilage, bones, meninges, and tongue” [1,2]. Other groups try to define fascial tissue, such as the Fascia Nomenclature Committee (2014): “The fascial system includes adipose tissue, adventitia, neurovascular sheaths, aponeuroses, deep and superficial fasciae, dermis, epineurium, joint capsules, ligaments, membranes, meninges, myofascial expansions, periosteum, retinacula, septa, tendons (including endotendon/peritendon/epitendon/paratendon), visceral fasciae, and all the intramuscular and intermuscular connective tissues, including endomysium/perimysium/epimysium [3].” The Federative Committee on Anatomical Terminology (FCAT) (1989), The Anatomical Terminology organization (1988), and the Federative International Programme on Anatomical Terminologies (FIPAT) (2011) consider the fascia as a tissue that wraps and separates: “a sheath, a sheet, or any other dissectible aggregations of connective tissue that forms beneath the skin to attach, enclose, and separates muscles and other internal organs [4].” Other authors propose definitions depending on the cellular level or function [5]. To fully understand a tissue, its function, and its relationship with other tissues proximal or distal, it is necessary to look at its embryological origin. The function of living tissue is generally influenced and often defined by its embryological derivation [6-9]. In the formed body, how do we decide where one tissue begins with respect to another when the same tissue has a dual embryological derivation? We need to broaden the vision of what is considered as a fascial tissue in light of the embryological information and its living derivative. For example, the dura mater has a dual embryological derivation. The dura mater of the forebrain area and caudal mesencephalon derives from the neural crests of the ectoderm, while the dura mater of the portion of the remaining midbrain and of the hindbrain derives from mesodermal cells [10]. The vessels that penetrate the meninges derive from the mesodermal leaflet [10]. Other examples include: the falx of the brain and the falx of the cerebellum, the tentorium of the cerebellum, the olfactory groove, and the medullary dura mater originate from the ectodermal leaflet, but the portion of the sigmoid, transverse, cavernous sinuses and the hypoglossal canal originate from the mesoderm [11]. In this case, while its origin is dual, its eventual function is unitary. The human body is not composed of separate layers, but of tissues seamlessly immersed in one another: this allows functional motion, life, and survival. Previously, we published articles that reviewed the embryology of the liquid fascia (blood and lymph) and solid fascia (bones, cartilage, smooth muscle fibres and involuntary striated muscle fibres) [1, 2, 4]. The article reviews the embryological evolution of skeletal muscle tissue and connective tissue, to affirm how the fascial system should be ideally conceptualized: an absolute anatomic functional continuum.

## Review

Connective tissue and voluntary skeletal muscle tissue

Musculature and connective tissue of the trunk and limbs

**Figure 1:**
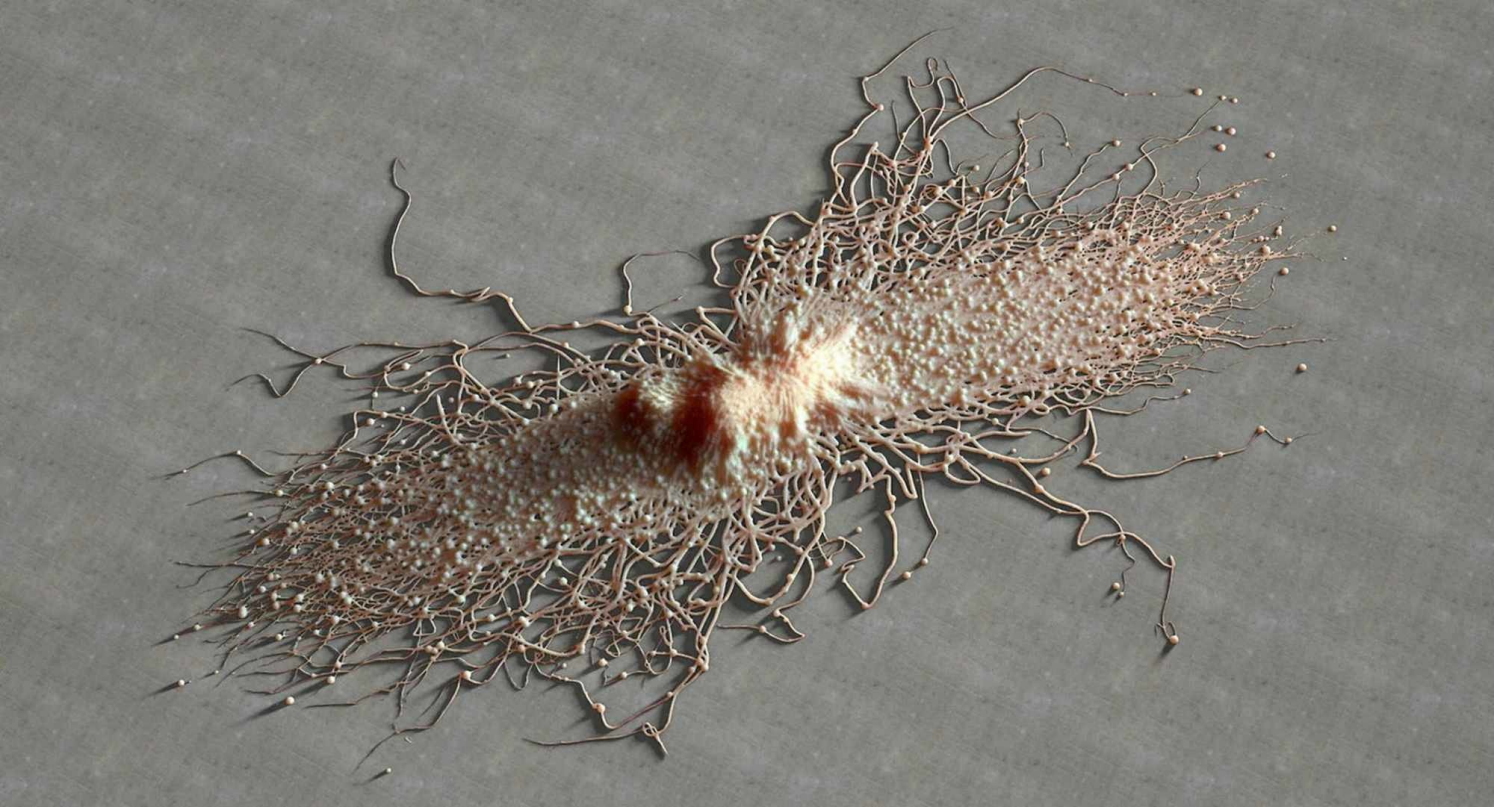
The image shows the three-dimensionality of a fibroblast by scanning electron micrograph.

**Figure 2:**
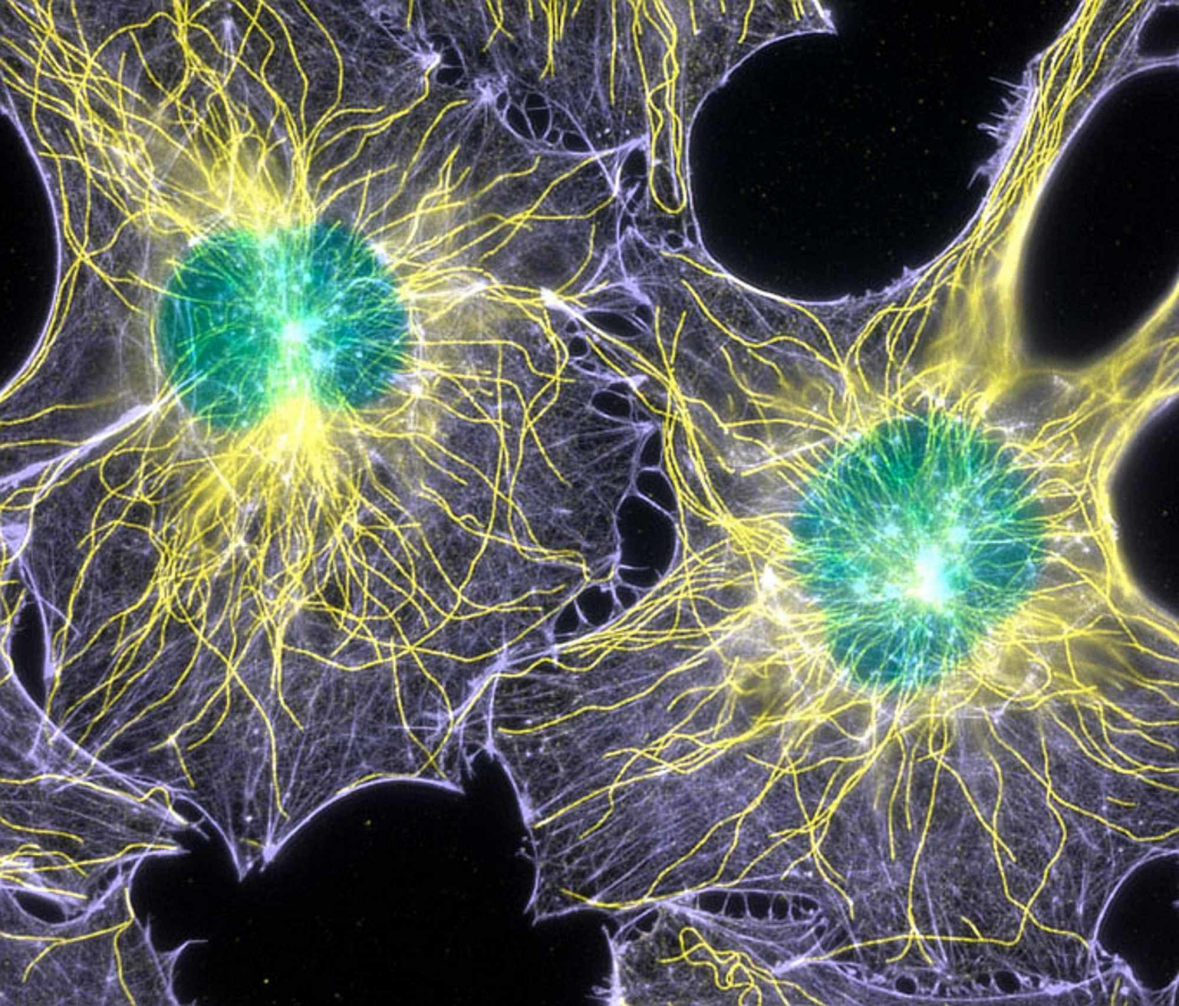
The image shows fibroblast cells with actin filaments and microtubules.

The human body consists of over 600 muscles, each with very complex myogenic development [12]. The somites derive from the paraxial mesoderm (somitogenesis) on approximately the eighth day of gestation. These are positioned laterally to the notochord and in a craniocaudal direction. The sclerotome emerges (eventually the meninges, ribs, neural arches, vertebral column) from the epithelial-mesenchymal transition of the ventral area of the somitic structure, while the dermatomyotome derives from the dorsal portion of the somite which maintains its epithelial organization for a longer period of time and becomes striated and smooth muscles, cartilage, dermis and angiogenic cells [13]. The dermatomyotome will give rise to the dorsomedial area (intrinsic muscles of the back), and a portion of the superficial ones (epaxial myotome or primaxial myotome), while the ventrolateral rippling of the dermatomyotome will develop the limb and ventrolateral muscles of the body (hypaxial myotome) [13]. At the level of the anterior and posterior occipital area, the myogenic primordial cells (mesenchymal cells) delaminate from the ventrolateral dermatomyotomic portion and migrate to those regions that will become limbs, tongue, diaphragm. A small part of the same mesenchymal migratory cells (future primordial myogenic cells), will return back towards the axial trunk once they reach their final destination [13]. The major percentage of mesenchymal cells will intrude into the lateral mesodermal dish (also referred to as abaxial or dorsal progenitor cells). The cells that will come back (known as In-Out mechanism) will form the superficial portion of the pectoralis major muscle and the latissimus dorsi muscle [13]. With the same mechanism, the perineal muscles will be formed. The connective tissues of the trunk and limbs will originate from the lateral plate of the mesoderm and from the somites [13]. Connective tissues guide myogenic cells towards their final destination and form. However, the connective tissue develops from the muscle tissue at different times (Figure 1, 2). An important notion that emerges from the study of embryo evolution is the ability of angiogenic cells to transform into striated muscle cells, passing through myoblasts, or smooth muscle cells, and passing through pericytes [13]. This further supports our view of smooth muscle integration as part of the fascial continuum [1,2]. The embryological origin of the musculature of the skull and part of the neck has a different path, as do the genes expressed to stimulate the growth of the craniofacial muscles.

Craniofacial and cervical musculature and connective tissue

**Figure 3:**
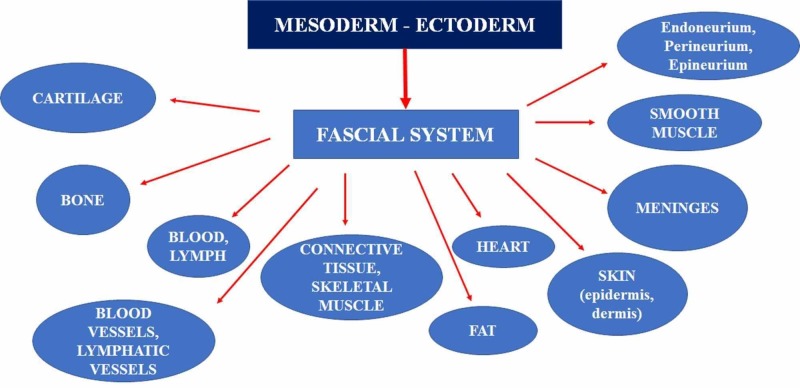
The figure illustrates the different embryological origins of the system or fascial continuum.

**Figure 4:**
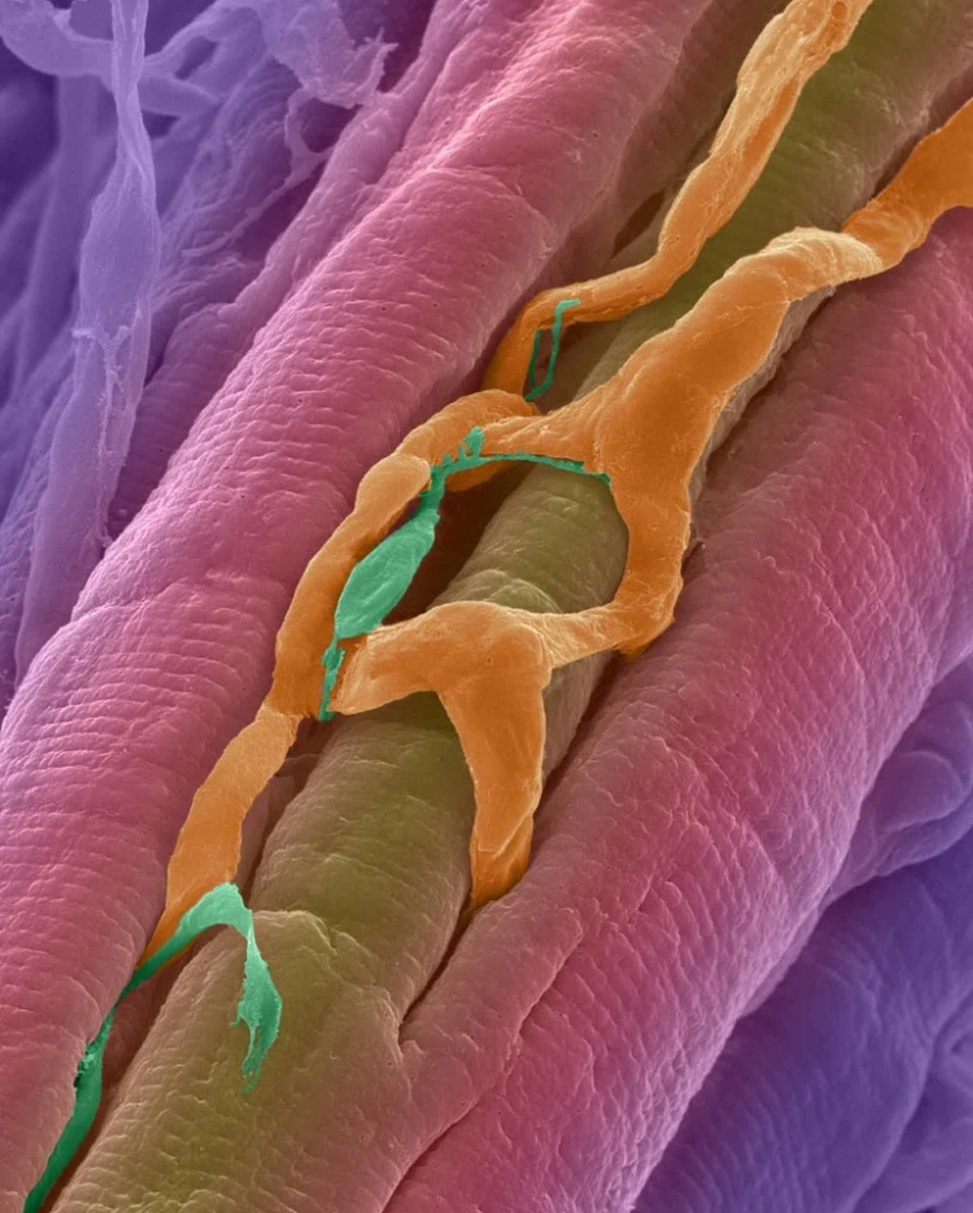
The orange reticulated in this scanning electron micrograph is made up of blood vessels intersecting the cardiac muscle fibers (in pink).

The embryological origin of the musculature of the skull and part of the neck has a different path, as do the genes expressed to stimulate the growth of the craniofacial muscles. Skeletal musculature is formed by myogenesis, stimulated by specific genes such as myogenic regulatory factors (MRFs: Myf5, MyoD, Mrf4, and Myogenin) and by the presence of paired box 3 (Pax3). For the development of the muscles of the facial area and of the high cervical tract, the presence of genes such as Myf5, MyoD and Pax3 are not essential, compared to the muscles of the trunk and limbs [13]. The T-box transcription factor 1 (Tbx1) gene is fundamental for the proper development of the muscles of the mandible and maxilla and is the same gene fundamental for the physiological development of some areas of the endoderm and of the ectoderm. The craniofacial muscles share the same developmental pathways as the heart, via genes such as Tbx1 and bicoid-related homeodomain transcription factor 2 (Pitx2), and the pharyngeal mesodermal origin [13]. There are about 60 muscles that act on the skull, the derivation of which are mesodermal cells anterior to the first somites. These latter areas are known as pharyngeal mesoderm and prechordal mesoderm [13]. The muscles resulting from these last two areas will act on the skull and partly on the anterior and posterior cervical tract. The muscles of the skull and upper cervical tract will come into contact with connective tissue that has part mesodermal and part ectodermal origin (neural crests coming from the dorsal area of the anterior neural tube) [13]. The pharyngeal mesoderm is divided into two areas in turn: the mesenchymal cells of the paraxial mesoderm, positioned laterally to the notochord and the neural tube; and cells of the splanchnic mesoderm, which have a strong epithelial tissue imprint. These two cell populations will form the core of the pharyngeal arches [13]. The pharyngeal mesoderm has a close relationship with the endodermal and ectodermal leaflet, the latter having a strong influence on muscle formation. The musculature of the cervical tract consists of about 80 muscles [14]. Part of the musculature of the neck will derive from the somites and the pharyngeal mesoderm, while part of the connective tissue deriving from the neural crests will influence the shape and function of some muscles of the craniocervical tract and of the shoulder: the trapezius muscle (anterior portion) and the sternocleidomastoid muscle (cleidomastoid portion) belonging to the cucullaris muscle group; the portion of the infrahyoid muscles; the buccal floor; the tongue, and the respiratory diaphragm [14, 15]. The cucullaris muscles share the genetic pathway of the cranial muscles and not of the trunk muscles [14, 15]. The neck and shoulder muscles and part of the back will contain mixed connective tissue, originating from ectoderm and mesoderm [14, 15]. The musculature of the trunk and part of the neck/shoulders as well as the connective tissue have different genetic pathways of development compared to the craniofacial musculature, the remaining part of the neck musculature, and the connective tissue of these areas [16]. Currently, it does not appear that the accounting for the different muscular genetic origin and the different embryological origin of the connective tissue results in different nomenclature of the fascia. From the data presented in the article, it is not possible to limit the respective embryological and genetic origins in the living organism as this would necessarily imply a difference in function. Regardless of its origin, the tissue is a coordinated functional continuum. Since the connective tissue and the meninges have a dual origin (mesodermal and ectodermal), and if this scientific evidence is not taken into account, the current assumption and usage of different nomenclatures and classifications fail. In another embryological example, the mesenchyme from the ectodermal leaflet is indispensable for the correct formation of the large vessels, in conjunction with the mesoderm [17]. The ectodermal leaflet must be taken into account to understand how to classify the fascial tissue correctly. Where is the border? Functional anatomy is a continuum, and not a series of segments or understanding of the continuum of function would cease. Presently, there appears to be confusion in the classifications, but only if the diversity of embryology is not taken into account. The classification proposed by our international research group, Foundation of Osteopathic Research and Clinical Endorsement (FORCE) is the only one in the breadth of scientific research that considers the embryological origins in pursuing a coherent classification of what it means for tissue to be considered fully as a fascial tissue [1, 2]. Other studies and future research will serve to better describe the function and with it, fascial classification (Figure 3, 4).

## Conclusions

The fascial continuum consists of solid fascia and liquid fascia. The embryological and genetic derivation of somebody areas where the fascial system is located has a dual origin: mesoderm and ectoderm. In the anatomy of the living organism it is not possible to distinguish a tissue of mesodermal origin from a tissue of ectodermal origin. For example, in the areas between the cervical tract, the back, and the shoulders; not even by developmentally tracking the cells that constitute it. The same is true for the meninges and the arterial system. To truly understand the fascial continuum, its function, and to determine a coherent nomenclature of the fascial system, it is necessary to take into account the differences in embryological origin. Not taking into account this scientific evidence, the assumption of different nomenclatures and classifications falls short and is incorrect. Our classification is the only one in the scientific landscape that takes into account the embryological information when classifying fascial tissue. Further studies and future research will serve to better delineate the function and fascial classification.
